# Text-Book of Pathology and Pathological Anatomy

**Published:** 1903-02

**Authors:** 


Text-Book of Pathology and Pathological Anatomy.—By Dr. Hans Schmaus, Professor in the Pathological Institute of Munich. Translated from the sixth German edition by A. E. Thayer, M. D., Instructor in Pathology, and edited, with additions, by James Ewing, M. D., Professor of Pathology in Cornell University Medical College, New York. In one octavo volume, 579 pages, with 351 illustrations, including 35 colored inset plates. Cloth, $4.00, net. Lea Brothers & Co., Publishers, Philadelphia and New York.

This book on pathology possesses a valuable combination of brevity, condensation and comprehensiveness embodying all the important principles and facts that should be brought before the student of pathology. The author has ingeniously compiled and condensed the present knowledge, amplified with a rich array of aptly chosen instances and references, which alone are sufficient recommendations to place this worthy volume on the shelf of every student.

Beginning with disorders of circulation its accompanying phenomenon and sequella, followed by retrogressive and progressive tissue changes, comprise the first 200 pages. Standing out with prominence in this section is the chapter on inflammation, which is aptly put with a careful avoidance of superfluity of statement and repetition of information. His definition of inflammation is "a condition of reaction exalted above the physiological normal pathologically altered and called forth by an external irritation."

Chapter 4 deals with congenital anomalies and deformities, well illustrated.

Chapter 5 gives a terse and comprehensive description of the various parasites.

Chapter 6 graphically includes the general bodily diseases from disturbed organic functions. And, alike, the succeeding chapters, following the same original style and ending with diseases of the skin, appendixed with a useful table of body measures and weights.

The plates and engravings are all that could be desired, highly descriptive and easy of comprehension..

The intrinsic worth of the volume to English speaking students and practitioners has been further increased by the work of the editor, Dr. James Ewing, Professor of Pathology, Cornell University, as well as by Dr. Thayer's very clear translation, throughout which the merits of the original have been admirably retained.

